# A review on additive manufacturing and its way into the oil and gas industry

**DOI:** 10.1039/c8ra03194k

**Published:** 2018-06-20

**Authors:** Merum Sireesha, Jeremy Lee, A. Sandeep Kranthi Kiran, Veluru Jagadeesh Babu, Bernard B. T. Kee, Seeram Ramakrishna

**Affiliations:** NUS Centre for Nanofibers and Nanotechnology, Department of Mechanical Engineering, National University of Singapore 2 Engineering Drive 3 Singapore 117581 vjbabu2002@gmail.com seeram@nus.edu.sg; Medical Materials Laboratory, Department of Metallurgical and Materials Engineering, Indian Institute of Technology Madras Chennai 600036 India; Department of Biotechnology, Bhupat and Jyoti Mehta School of Biosciences, Indian Institute of Technology Madras Chennai 600036 India; Singapore Institute of Manufacturing Technology, A*STAR Singapore-138634 babu@simtech.a-star.edu.sg; Downhole Equipment New Product Development Department, Artificial lift Singapore Integration Center, Schlumberger Oilfield (S) Pte. Ltd. 7 Benoi Crescent Singapore 629971

## Abstract

In the near future, the oil and gas industry is poised to become one of the greatest sources of revenue generation across the world. The adaptation of scalable manufacturing technology, commonly known as additive manufacturing (AM) in the oil and gas industry, offers huge potential to transfigure the way high quality 3D objects are designed, manufactured and distributed. The adoption of AM technologies in this sector also allows a high degree of freedom of design and could exponentially reduce the time taken for the product to reach the market. In this arena, AM can be a method of producing lower volume and highly efficient intricate products with various materials like polymers, metals, ceramics and their composites. Although AM has been around for several years, its adoption in this sector has been slow and limited. As it is in the initial stages, rigorous research needs to be done to standardize the materials and manufacturing process. In addition, there is a particular need to end the requirement of a finishing procedure. Continuous and significant growth has been seen since the beginning and the successful outcomes until now allow for optimism that AM has a significant role in the future of manufacturing. This review will mainly focus on ongoing efforts to bring widespread adoption of AM into highly regulated industry *i.e.* oil and gas, and will also identify future perspectives in this area.

## Abstract 2

The Schematic representation of a Stereolithography (SL) scalable manufacturing technology called additive manufacturing (AM) in oil and gas industry.

## Graphical abstract

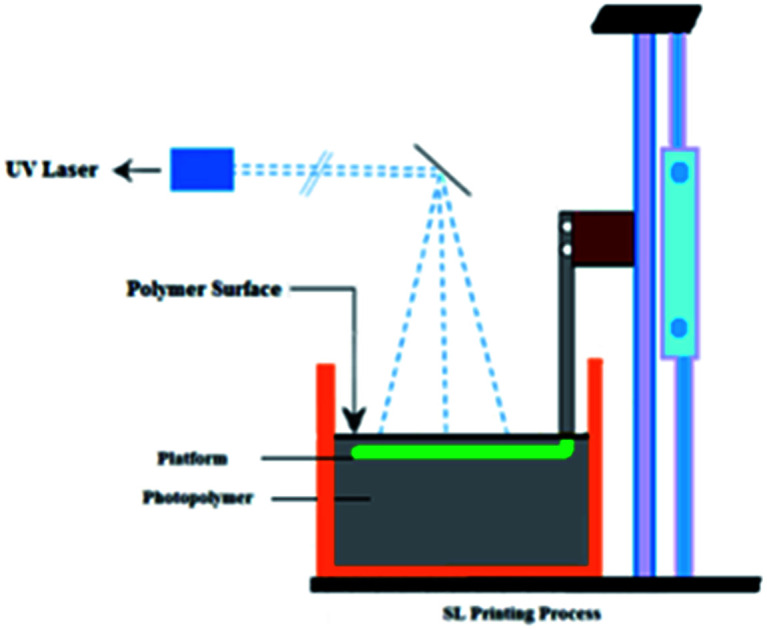

## Introduction

Additive manufacturing (AM), or 3D printing, builds objects layer by layer using 3D modelling data. AM has been explored from rapid prototyping to tooling that leads to direct production. More importantly, AM can be used to integrate with CAM (computer-aided manufacturing), CNC (computer numerical control) and CAD (computer-aided design) for 3D printing objects.^1–4^ AM is applied everywhere from biomedical applications to aircraft design and is being slowly explored for applications in the oil and gas industry. The materials used in AM include polymers, metals, ceramics and their composites; however the materials for AM are still limited. For instance, in some cases CNC machining is needed as, sometimes, the dimensions of the spare parts to be built can be larger than available AM printers can cope with. Rapid prototyping may not be a good answer for all instances as CNC machining could also be required.^3–5^ In the past few years, AM has played a key role in the oil and gas industry by promoting the engineering nozzles produced by the GE company.^6^ Although AM has significant opportunities in the oil and gas industry, the truth is that real companies have become slower to take them. However, major oil and gas service companies have invested in AM and have completed some successful pilot projects.^7,8^ AM is potentially capable of enabling the design of products with complex structures with reduced cost and waste and could also reduce the overheads associated with documentation and production planning.^9^ AM technology produces parts with fewer materials compared to conventional technologies and provides a quick response to demand for spare parts.

## Industrial additive manufacturing

**Fig. 1:**
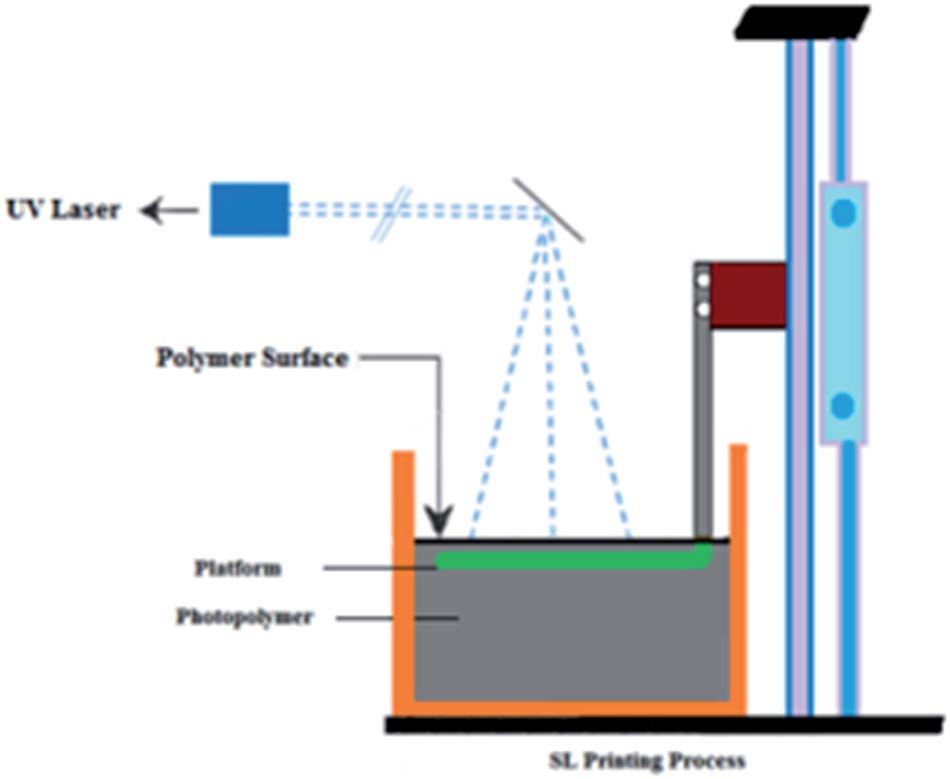
Schematic representation of the SL printing process.

Several AM methods have been developed and introduced into the industrial market, with manufacturers like Electro-Optical systems, Optomec, 3D Systems, Stratasys, AeroMet, Precision Optical Manufacturing, *etc.*^10^ Stereolithography (SL) and digital light projection (DLP) are the two VAT polymerization methods. SL is an AM technique used to create parts from 3D computer-aided design software and translated to an STL file in which CAD data is sliced into thin 2D mathematical equations. This input data is transferred to an SL (AM) system containing a VAT liquid-based UV-curable photopolymer. The printer begins to form one layer at a time, in which the UV laser beam is guided onto the liquid resin by controlled mirrors and the laser traces then solidify the cross-section of the layer. Once a layer is complete, the process will repeat until the part is complete, then the platform moves down and makes space for the next layer. A blade moves across the surface to ensure a smooth surface layer. The laser continues to trace and form each layer atop the previous layers, building from the bottom up. The completed part will be removed and separated from the platform and the part is cured in an ultraviolet oven. SL has become an excellent economical choice for rapid prototyping. Multiple material SL is also available, which changes the materials used for subsequent layers. A wide variety of industries apply SL including chemical engineering, entertainment, packaging and sporting goods,^11^ automotive & aerospace^12^ and biomedical,^13^ which will be discussed in the applications section. A schematic of the SL printing process is shown in Fig. 1.

Digital light processing is an upcoming method that uses illumination of resin. This technology is similar to SL but the advanced feature DLP has is a digital mirror device, an array of millions of self-rotated mirrors, and by projecting, the 2D pixel pattern can complete entire layers at once which reduces the build time.^14–16^

**Fig. 2:**
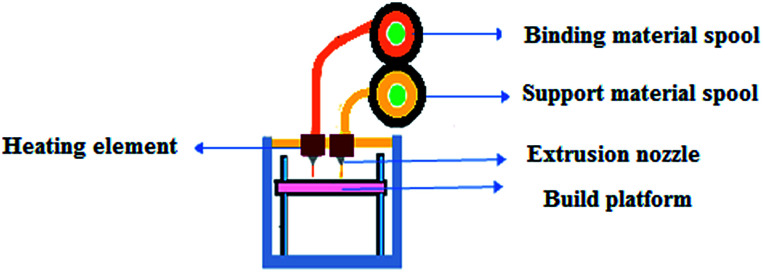
Schematic representation of the fused deposition process.

Fused Deposition Modelling (FDM) involves a filament made of thermoplastic polymer which is melted by heat and extruded through a nozzle to form layers on the building platform.^17^ The available filament spools have a diameter of 1.75 mm or 3 mm. The filament is unrolled from the spool into the extrusion head, which is a similar mechanism to that of a hot glue gun (Fig. 2). The advantages are cost-effectiveness, no required chemical post-processing and less expensive machinery.^1,2^ The disadvantages are the low resolution compared to other AM processes and that if a smooth surface is needed, the finishing process is very slow for large complex parts. To save time, some models permit a fully dense mode and a sparse mode that saves time but with a reduction in the mechanical properties.^18^

There are different types of powder bed fusion printing methods like electron-beam-melting (EBM), selective-laser-sintering (SLS), selective-laser-melting (SLM) and direct-metal-laser-sintering (DMLS). SLS is a 3D printing process in which powder is sintered with a carbon dioxide (CO_2_) laser beam. The chamber is heated up to just below the melting point of the substance. The laser precisely fuses or melts the particles at the surface layer by layer. A piston controls the layer thickness each time a layer is finished to ensure each layer has exactly the same thickness. Polymers that could be used are acrylic, styrene and nylon and metals such as copper and composites such as polyamide with fiberglass *etc.* can also be used.^19–22^ In DMLS, metals and alloys are used to build the parts *e.g.* steel and cobalt chrome. SLM is an advanced form of the SLS process where full melting of the powder bed particles takes place using one or more lasers. During all the above-mentioned processes, high temperatures and controlled atmospheres (using inert gases) are required. These inert gases can prevent oxidation and leave the mechanical properties unchanged.^23^ EBM involves heating the powder with an electron laser beam powered by a high voltage, typically 30 to 60 kV. This process is very similar to SLS.

EBM has a high energy density compared to SLM, but EBM allows thicknesses even higher than 100 μm for each layer and a large particle size distribution.^24^ A Ti_6_A_l4_V hexagonal cone-shaped honeycomb rotor with pore channels can be used for oil & gas separation.^25^ There are other compatible materials with the EBM process such as nickel superalloys (Inconel 718 & 625, Rene142, and CMSX4), cobalt superalloys, copper, stainless steel and CoCrMo.^24,26–30^ The ability to cure the ceramic suspensions deposited on a glass substrate, where the building portion is not fully immersed in the liquid feedstock, leads to reduced procedure costs and utility of raw materials.

The Massachusetts Institute of Technology (MIT) fabricated highly complex structures of any dimension with unparalleled flexibility.^31^ In their method, a thin layer of material is spread over the powder bed and a liquid droplet of a binder material is dropped through the print head over the surface. The print head then prints the binder with the layer, causing the bonding of end-to-end powder particles. The powder bed station is then lowered by a certain height and a fresh layer of powder is spread over the previously bonded layer. The process of lowering the powder bed platform, spreading a new layer over the previous layer and printing with the binder material continues until the fabrication of the 3D object is completed. 3D printing involves the use of solvents and binders. 3D printing has proven its capability in both industry and the biomedical sector by fabricating diverse materials. Materials such as polymers (starch, cellulose, PLGA, PCL, PLA), ceramics (Al_2_O_3_), metals (steel) and shape-memory alloys that demonstrate exceptional geometries can be created using 3D printing.^32–36^

MultiJet Printing is an inkjet printing process that uses piezoelectric technology to jet the material onto a build platform layer-by-layer. In this process, the droplets of material are initially deposited from the print head onto the surface and solidify to make up the first layer. Further layers are built on top of the previous layers. The material layers are then cured or hardened using UV light.

**Fig. 3:**
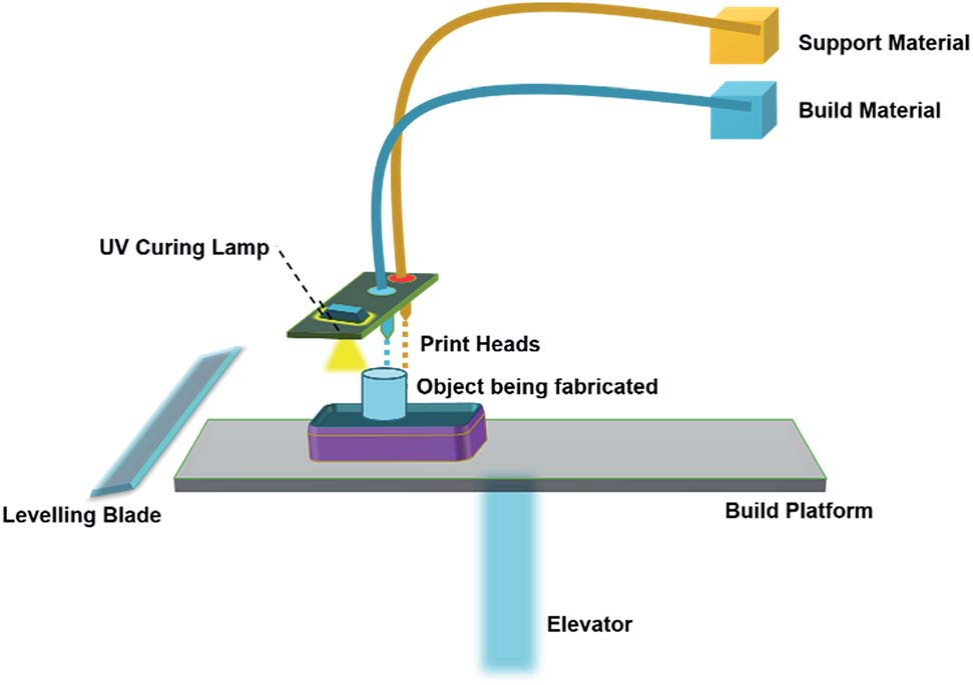
Schematic representation of the material jetting process.

A schematic of material jetting is shown in Fig. 3. Several investigations have been carried out on two modifications of ink-jet printers, namely Continuous Inkjet Printing (CIJ) and Drop-On-Demand (DOD). The unique difference between CIJ and DOD is the timing of droplet generation. In DOD, droplets are generated when required, whereas in CIJ, the droplets are generated by breaking up the continuous stream of droplets through an ejection nozzle. In all AM technologies, material jetting is the only technology that offers highest *Z*-direction resolution with layer thicknesses as low as 16 μm. Materials such as ABS, polyamide, PLA and their composites are commonly used for printing 3D objects by CIJ and DOD.^37–39^

**Fig. 4:**
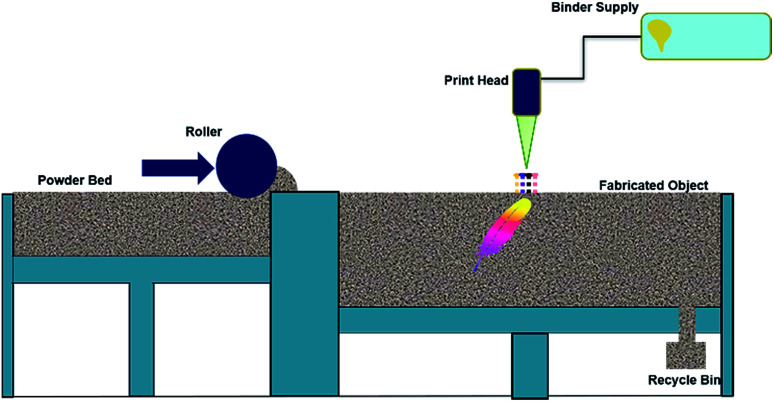
Schematic representation of binder jetting and the components used for the part fabrication process.

Binder jetting builds 3D objects through inkjet printing of a binder into a powder bed of a raw material without using any external heat sources. In this process, a fine powder is layered onto a build platform, and then a liquid adhesive agent is carefully applied through inkjet print heads to adhere the particles together. The build platform is made to lower by a pre-fixed distance and the next layer of powder is laid on top of it. The next layer is then printed and is bonded to the previous layer by the jetted binder. By repeating the process of laying out powder and bonding, the parts are built up in the powder bed (Fig. 4). This technology is capable of printing a variety of materials that are available in powder form, including materials like metals, polymers, and ceramics.

As no external heat source is required in this technology, the parts built are free from residual stress, which significantly reduces the need for secondary post-processing operations. Since the 3D objects are built by gluing the particles together, the mechanical properties obtained by this technology are very limited and are generally not recommended for any structural applications.^39–44^

**Fig. 5:**
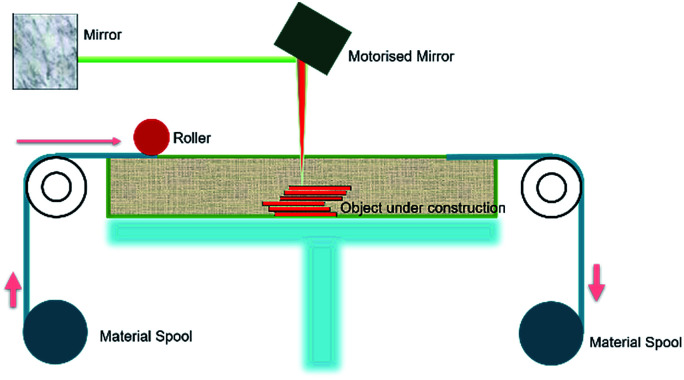
Schematic diagram showing the sheet lamination process.

Sheet lamination, also known as Laminated Object Modelling (LOM), manufactures objects and prototypes by cutting, sequentially laminating, and bonding. LOM works on a principle where thin adhesive-coated metallic sheets or layers of plastic are bonded together using ultrasonic welding and shaped by a laser cutter. A schematic of the sheet lamination process is shown in Fig. 5. Since the process involves solid state bonding and additional adhesives are used, the material is not required to reach its melting point for the bonding to occur. A variety of materials can be manufactured using sheet lamination which includes paper, ceramics, metals (aluminum, stainless steel, copper and titanium), plastics, fabrics, synthetic materials and composites.^45^

## Potential benefits of AM

Various technological methods exist from printing liquefied polyamide with “desktop printers” to laser sintering ceramics, for example, which can be found in the aviation sector. These techniques are not intrinsically new but have been used for years in prototyping.^45^ The trade-off in manufacturing that exists is between the major dimensions of flexibility (responding to quick changes and offering a broad range of product variants) on the one hand and efficiency (lead time and variable cost) on the other. Achieving flexibility and efficiency is not possible simultaneously in manufacturing systems. Hence, they must be subsequently optimized for a single specific objective to achieve a balance between job shop and flow shop production. The enormous rise of AM is based on the option of rapid tooling (higher level of flexibility on production and tools). AM can be used as an automatic set-up which substitutes for labour in job shop manufacturing. AM offers the customer a broad product range, specified products or more concise product life cycles.

**Fig. 6:**
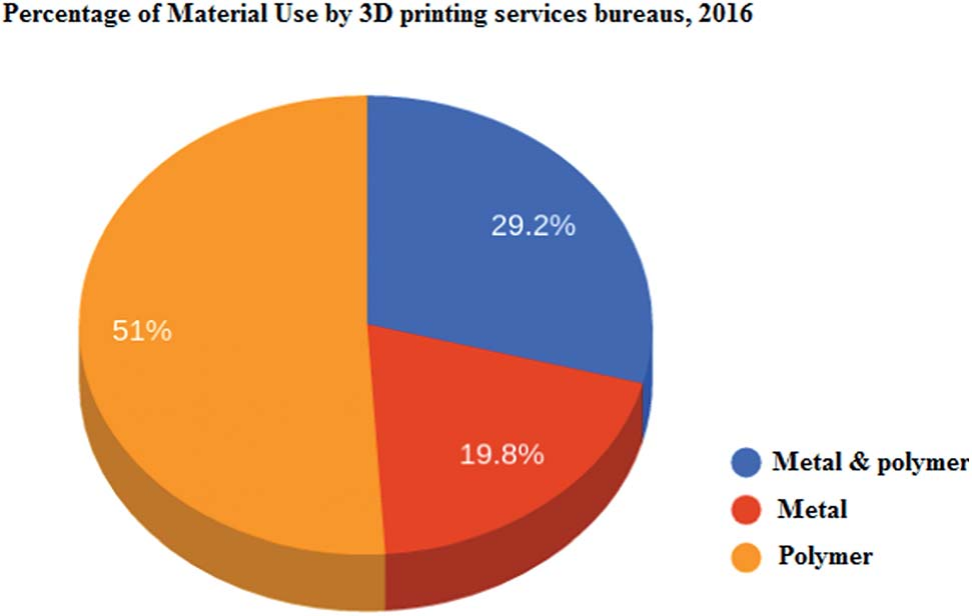
Percentage of materials used by 3D printing services bureaus, 2016. Source: (Wohler’s Associates) http://gfxspeak.com/2017/04/04/wohlers-printing-industry/.

The supply chain efficiency of AM reduces the costs of storage and transportation of the raw materials and mid-process and end-usable parts. Production of the spare parts inventory on demand without the need for setting up and tooling could be a contemporary solution in supply chain management^46^ (Waller and Fawcett, 2014) (Fig. 6).

The time taken for the whole cycle of manufacturing to marketing in AM is reduced due to having few design and prototyping requirements, there being more anticipated factory loads, manufacturing tools not being required and having no factory setup times. There is a lot of scope for both designing and redesigning the prototypes and parts without adding to the production costs. The time and material requirements to build a part are estimated absolutely before loading on to the machine and having the ability to read CAD files improves the planning, tracking and measuring of the volume and capacity at any given moment.^47^ Mass customization of AM allows for cost-efficient conversion from conventional mass production to new areas of mass customization and the capability to employ multiple designs on the same machine could enable the manufacturing industry to move from mass production in factories to mass customization with distributed manufacturing. Using materials ranging from polymers to metals and even human cells, AM constructs complicated products of several varieties that can be made to exact customer specifications. Designate Products, a division of 3D Systems, additively manufactures custom-designed prosthetic human body parts, such as legs, with sophisticated features such as body symmetry, locking knees and flexible ankles. During the development process, customers have the choice to select the material to model. AM also influences the cost, for example, the cost of prosthetic legs is usually $60 000, which drastically reduces to $5000 with AM, which also provides great features which are not possible in existing prosthetic legs.^48^

**Fig. 7:**
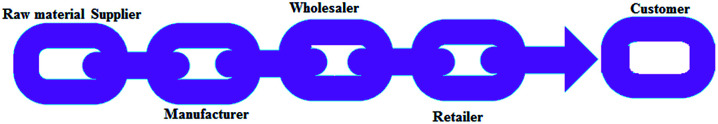
Schematic of the general supply chain structure.

The economic potential of AM is as a manufacturing tool-free system, which produces separate parts and small batches without fixed time and bulk resources. This confirms the exclusion of material and capital input into tool production. In addition, the need for a capital-intensive supply of specific manufacturing amenities and specialists or experts is reduced. The infrastructure of the AM is “Print on Demand” and this results in the flexibility of separate product development and production, leading to new models that focus on service either within product development or offering manufacturing resources (Fig. 7).

BCG calculated that AM marketing in 2015 had grown to approximately $5 billion. They estimated that it would grow at a compound annual growth rate of almost 30% through 2020, achieving more than a threefold increase in size. If AM processes were adopted for approximately 1.5% of the total addressable manufacturing market by 2035, the AM market would exceed $350 billion. We expect metal-based AM technologies to capture an increasing share of the total AM market. Ecological sustainability of AM explicitly includes the reduction of hazardous waste by location-independent manufacturing. According to expert reports, the energy savings would be approximately 50% or even more in applications where AM is competitive.^49^

## Additive manufacturing applications in oil and gas

AM technology has gained significant traction in the aerospace sector, where its potential advantages are most clearly realized. These include being able to manufacture small quantities of specially designed parts that are lighter, have more complex functional geometries and are made of expensive high-performance materials such as nickel superalloys that conventional manufacturing technologies are relatively less efficient at producing.^50^ Specially designed parts optimized for AM could provide weight savings, cutting down the cost of operating the aircraft significantly. A re-designed titanium AM seat-belt buckle was estimated to be 45% lighter than its conventional aluminum counterparts without compromising its strength, leading to fuel savings of over 3 000 000 liters over the course of an Airbus A380 aircraft’s service lifetime.^51^ Besides these manufacturing performance advantages, the vertically integrated supply chain model adopted by many of the large aviation OEMs in a tightly regulated industry render on-demand additively manufactured parts more cost-effective, given the immense number of high-value, precision engineered components needed to assemble the aircraft and the fact that most parts tend to be required in small numbers.^52^ Design optimization for AM could also lead to parts having fewer components, cutting down on the number of external vendors involved, thereby decreasing the need for costly inventory management, quality control inspections, documentation and regulatory approval throughout the supply chain.^53^ In terms of the active deployment of AM technologies in the aerospace sector, AM has been used to produce turbine housings, helicopter engine combustion chambers, gas turbine exhausts, turbine vanes with internal cooling channels, jet engine fuel nozzles, bracket connectors, complex gear casings, structural hinges, transmission housings and many other components using a variety of materials and AM processes.^54^ In contrast, the oil and gas industry has been much slower in embracing AM for directly manufactured end-use products, with rapid prototyping of tools and complex models finding favor instead. The use of AM prototypes in the planning phase of the Stones Deepwater Project helped to bring down the costs associated with installing foam blocks into a buoy for one of the deepest water installations and demonstrating the feasibility of the operation to expedite the obtaining of approval from regulators.^55^ Similarly, the use of AM has allowed GE oil and gas to reduce the product development time of a burner for a NovaLT16 gas turbine by enabling the rapid prototyping of design concepts and accelerating validation testing, resulting in savings of over 50%.^56^

**Fig. 8:**
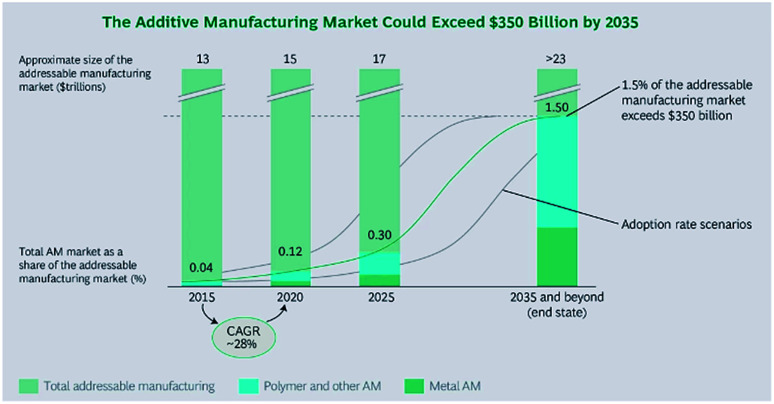
BCG analysis, in which data covers the AM market across the value chain. The figures presented relate to the middle adoption scenario.

Where AM might have the most disruptive impact on the oil and gas industry may be more the supply chain management^57^ than the AM part performance or production cost advantages. With oil and gas assets being deployed in increasingly isolated regions for longer durations, there is a correspondingly larger likelihood of essential parts breaking down, becoming obsolete due to technological changes or changes to standards or going out of production before the asset is decommissioned. The small numbers and short life-cycles of these parts relative to the component present a unique challenge in inventory management and further strengthen the case for the deployment of AM.^58^ Obsolete parts can be reverse-engineered digitally and additively manufactured on demand, leading to greater asset longevity in cases where the faulty part of a critical component can simply be 3D printed and replaced like-for-like (Fig. 8).

Likewise, the democratization and delocalization of just-in-time (JIT) manufacturing afforded by AM could lead to significant cost savings in the form of production lead time, inventory management and transportation of replacement parts, reducing asset downtime.^59^ In this regard, the Rotterdam Additive Manufacturing Lab (RAMLAB) has pioneered the use of robotic arm wire-arc AM together with CNC milling to manufacture a prototype 400 kg, triple bladed propeller out of a corrosion resistant nickel–aluminium–bronze alloy as the world’s first classed and approved AM ship propeller. Their innovation paves the way for the rapid fabrication of replacement parts for ships arriving at port in need of repairs at any port around the world without the need to order, manufacture and deliver the replacement to the ship, saving the asset owners significant up-front and downtime costs.^60^ There can also be a case made for AM capability to be installed directly on board oil rigs and ships in order to provide instant availability of replacement parts for assets located in inaccessible areas or in cases where delivery is impracticable.^61^

**Fig. 9:**
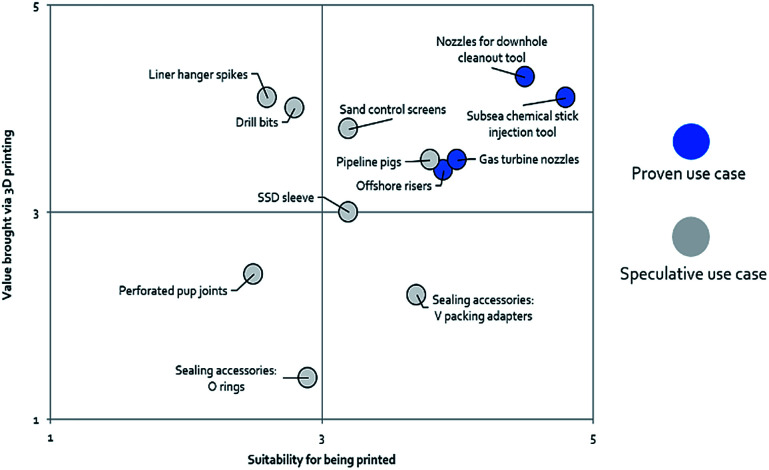
Use cases of AM components in the oil and gas industry.

According to Lux research, there are twelve speculative cases in which the oil and gas industry can potentially use AM for the fabrication of end-use products, as shown in Fig. 9. According to their scoring methodology, based on the components’ feasibility to be printed along with their value proposition, high value manufactured components such as downhole cleanout tool nozzles, offshore risers, gas turbine nozzles and subsea chemical stick injection tools already have proven use cases.^62^ The focus on the selection of potentially lucrative use cases appropriate for AM appears to be driven by the principle of “high geometric complexity–low volume”, where the savings in terms of customization and reducing material wastage incurred *via* subtractive manufacturing play into the economics of AM.^63^ Such a part could include molds to fabricate complex replacement fixed cutter drill bits, which would have taken weeks to prepare by casting a template, removing displacements, and fixing individual teeth to the body that could alternatively be accomplished relatively simply through AM.^64^ In fact, GE oil and gas has already pioneered the use of AM for small final parts in its oil and gas technologies such as valves and turbomachinery parts.

**Fig. 10:**
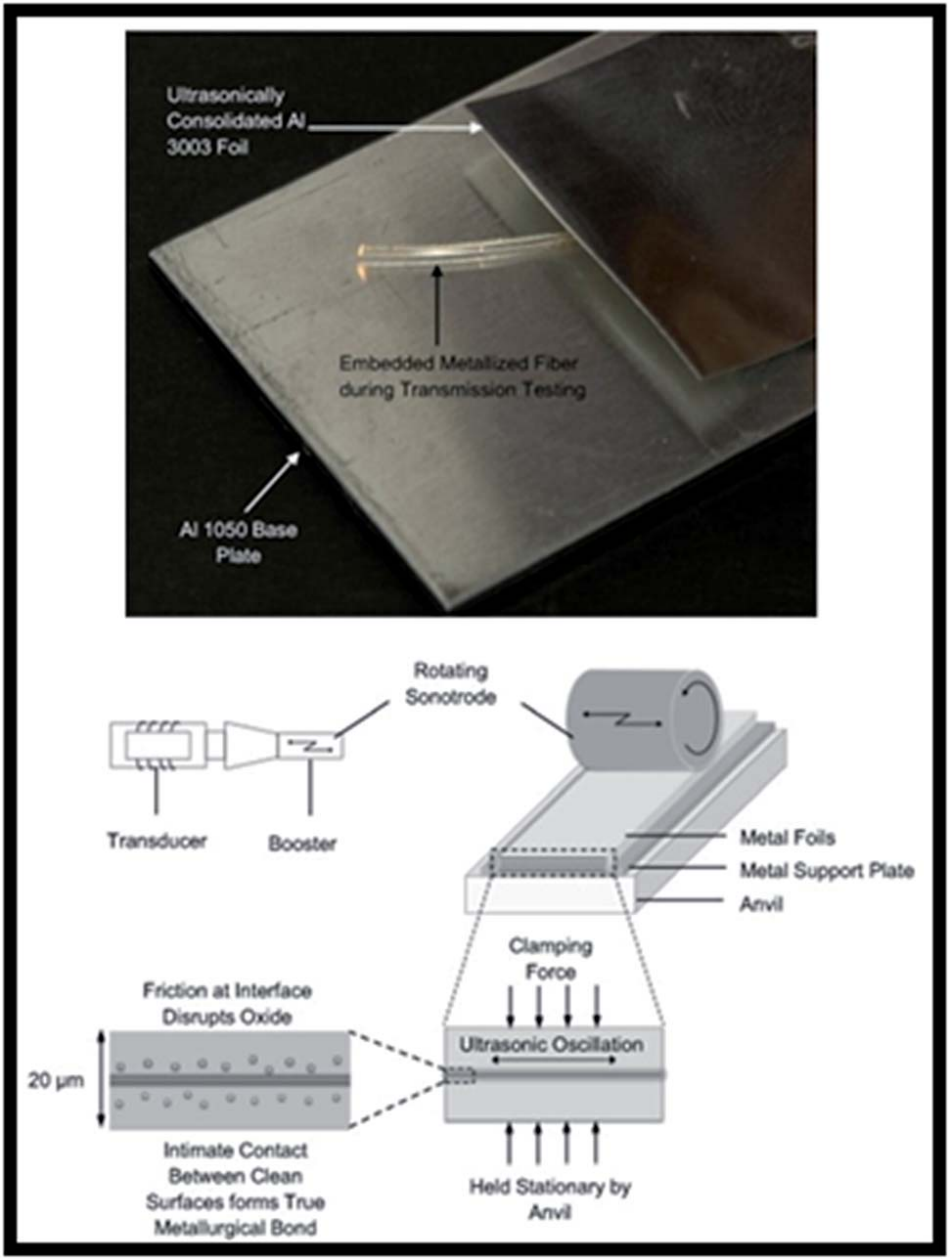
The incorporation of a fiber optic cable in an additively manufactured structure (top) and processing schematic (bottom).

A use case for AM that is receiving much research attention lately is the embedding of sensors for structural health monitoring of oil and gas assets during the AM fabrication process.^65,66^ Fiber optic sensors implemented in the corrosion monitoring of pipelines^67^ have been successfully metallized and then incorporated into an aluminium matrix structure through the use of solid-state ultrasonic AM while retaining full fiber sensor function, as shown in Fig. 10.^68^ Similar work done by Maier *et al.* demonstrated that optical fiber sensors containing fiber Bragg gratings could be embedded in a polymer component additively manufactured using powder bed fusion by interrupting the build process to insert the fiber carrier.^69^ The design freedoms permitted by AM technologies not only allow the placing of sensors in the bulk of the structure to detect defects and monitor health but they also allow the embedding of various functional systems like circuits, energy harvesting devices, piezoelectric actuators, *etc.* in pre-designed voids which can then be encapsulated.^70^ There has been much research done in this field for various AM methodologies^71–79^ which validates the enormous potential for the development of these “smart” structures.

One of the more frontier potential AM use cases in the oil and gas industry involves an added dimension of environment or time induced shape responsiveness known as 4D printing.^80^ Although the implementation of this technology is still not so clear, the oil and gas industry is excited to initiate collaborations with the company Geosys Tech that can develop peristaltic pipes.^81^ While AM offers many opportunities for the oil and gas industry, there remain many technological and regulatory hurdles to overcome before it becomes a viable commercial alternative to conventional manufacturing.

Perhaps the most apparent limitation of AM is its prohibitive cost in manufacturing end-use parts. As previously described, the economics of AM favor “high complexity–low volume”, just-in-time production, however, 99% of manufactured parts do not require customization^82^ and benefit tremendously from the economies of scale, rendering AM uncompetitive for scale manufacturing. The cost of metal AM fabricated components is largely dominated by the platform cost, with material, power and post-processing costs further adding to the expenses.^83^ Machine utilization is therefore maximized when the number of parts to be manufactured simultaneously on a single build tray is maximized. AM platform costs could be driven down as the technology matures and there are more adopters of AM, likewise the cost of the metal powder. Build speeds and build tray sizes could also improve, along with new part designs that exploit the advantages of AM, allowing better utilization of the machine to make AM more cost competitive. To this end, there already seems to be progress with AM platform costs already coming down 51% between 2001 and 2011,^84^ and the development of faster platforms such as Toshiba’s laser metal deposition machine, which is capable of AM at 10 times the speed of powder bed fusion technology.^85^

Besides the prohibitive cost associated with AM, another major risk hindering its adoption lies in the inherent variability of the process leading to uncertainties. These variabilities could include the powder feedstock characteristics between batches and suppliers, powder degradation during storage and recycling, material properties after sintering/melting, quality control of the built part, anisotropy of the part based on build orientation, differences in AM platforms and systems, consistency between prints, design validation of parts optimized for AM, build order and support structure, post-processing approaches, *etc.* As a revolutionary technology, there also remain many uncertainties with regard to the health and safety of the process, especially considering the nanometer sizes of the metal particles used. Issues also arise from uncertainties in the intellectual property ownership between designers, customers and AM service providers related to the digitized designs of existing parts, as well as the reverse engineering of in-service parts by third parties without licensing IP from the OEM.

**Fig. 11:**
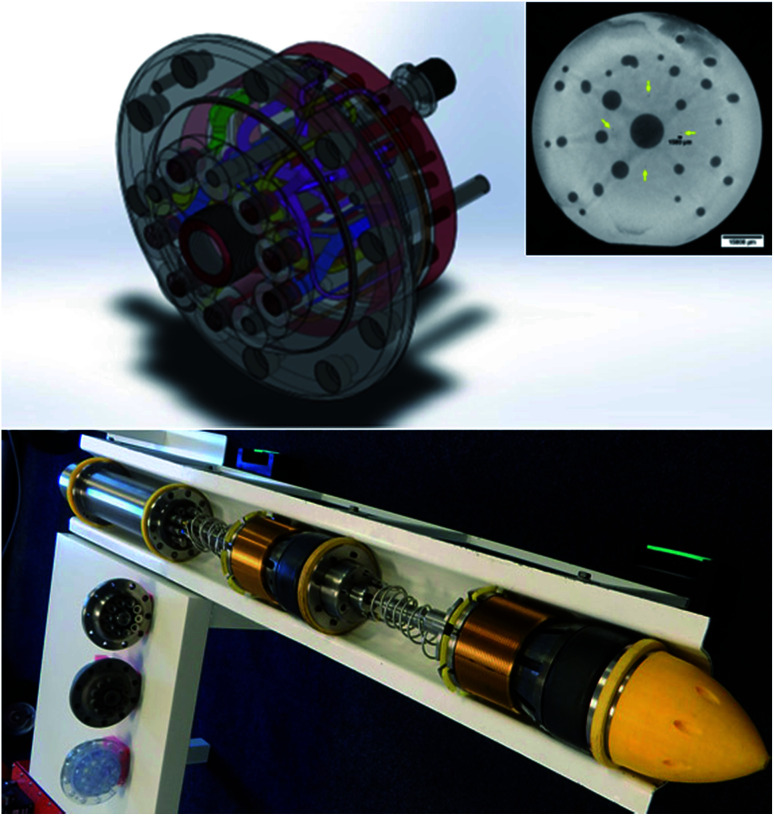
The CAD design of the gateway manifold with complex internal channels (top), cross section showing the fine resolution and small feature sizes enabled by additive manufacturing (inset) and the product at each stage along the manufacturing process and deployed in the assembled pipeline isolation tool (bottom).

There also needs to be a robust regulatory framework to improve confidence in AM manufactured parts across the industry. In this regard, initial steps have been taken by regulatory bodies and government agencies toward providing assurance of an AM part’s fitness-for-purpose in critical applications. One example is the certification of an additively manufactured titanium 6′′ gateway manifold for use in a pipeline inspection tool manufactured by the Safer Plug Company in accordance with the newly established industrial standards STM F2924-14: standard specification for AM titanium-6 aluminum-4 vanadium (Ti–6Al–4V) with Powder Bed Fusion. The gateway manifold is to be included in an assembly for a suite of pipeline isolation tools, which will include the world’s smallest tool suitable for six-inch diameter pipework. Fig. 11 shows the complex internal channels within the gateway manifold as depicted in the CAD model, a design that can be rapidly prototyped and economically realized through AM technology. The cross-section of the gateway manifold, also shown in Fig. 11, highlights the fine resolution that can be achieved through powder bed fusion technologies, with features such as internal channels as small as 1 mm in diameter made possible. According to the Safer Plug Company, the design changes have resulted in better performance, and the company is, at the time of writing, undertaking type approval certification to be able to produce gateway manifolds on demand.

The development of recognized standards goes some way towards establishing confidence in the AM process and provides a framework for early adopters of the technology to assure clients of the safety and reliability of the parts, especially for critical applications.^86^

## Conclusions

3D printing for oil and gas applications is in its beginning stages. Based on the specific dynamics of the global oil and gas market in the next decade, the need for the adoption of AM by the oil and gas industry is mainly because of its ability to reduce costs by cutting away unwanted material as 3D printing makes an exact shape layer by layer. This leads to reduced manufacturing time as well. Identifying the scope of applications by evaluating the current prototype list and widening the screening of components on oil and gas facilities are subject to strict testing requirements. There is significant work required for testing and approvals can be done on a product range rather than single printed products. The opportunity in the oil and gas industry is not just in the machinery parts, but also in the products that support oil and gas operations, which have lower critical importance, and hence lower quality requirements. Industry landscape, technology and quality issues/approvals are the factors that could determine the future adoption of 3D printing in oil and gas.

## Conflicts of interest

There are no conflicts to declare.

## Supplementary Material

## References

[cit1] Noorani R. , Rapid Prototyping: Principles and Applications, Wiley, 2006

[cit2] Cooper K. G. , Rapid Prototyping Technology: Selection and Application, Marcel Dekker, Inc., New York, NY, USA, 2001, ISBN: 0824702611

[cit3] Kruth J. P. (1991). CIRP Ann..

[cit4] Wohlers T. , Manufacturing Engineering, 2012, vol. 148, pp. 55–56

[cit5] Zhong A. , Ornelaz R., Krishnan K. and Offshore Technology Conference held in Houston, 2017, 0TC-27540-MS

[cit6] http://www.bloomberg.com/news/2013-05-14/how-3-d-printing-could-disrupt-the-economy-of-the-future.html

[cit7] Bomen, http://www.downstreamtoday.com/news/article.aspx?a_id=49720&Aspx.AutoDetectCookieSupport=1, 2015

[cit8] J. T, JPT, 2016, http://www.mydigitalpublication.com/article/3D_Printing_In_The_Oil_Field_Kicks_Production_Mode/2537582/322123/article.html

[cit9] Wohlers T. and Caffrey T., Wohlers Report 2014: 3D printing and additive manufacturing state of the industry; Annual worldwide progress report, 2014, pp. 1–10

[cit10] Guo N., Leu M. C. (2013). Frontiers of Mechanical Engineering.

[cit11] Calignano F., Manfredi D., Ambrosio E. P., Biamino S., Lombardi M., Atzeni E., Salmi A., Minetola P., Iuliano L., Fino P. (2017). Proc. IEEE.

[cit12] Lopes A. J., MacDonald E., Wicker R. B. (2012). Rapid Prototyp. J..

[cit13] Scarfe W. C. (2015). Oral Surgery, Oral Medicine, Oral Pathology, and Oral Radiology.

[cit14] Choi J.-W., Wicker R., Lee S.-H., Choi K.-H., Ha C.-S., Chung I. (2009). J. Mater. Process. Technol..

[cit15] Han L.-H., Mapili G., Chen S., Roy K. (2008). J. Manuf. Sci. Eng..

[cit16] Liska R., Schuster M., Inführ R., Turecek C., Fritscher C., Seidl B., Schmidt V., Kuna L., Haase A., Varga F., Lichtenegger H., Stampfl J. (2007). J. Coat. Technol. Res..

[cit17] Brian N. T., Robert S., Scott A. G. (2014). Rapid Prototyp. J..

[cit18] Morvan S., Hochsmann R., Sakamoto M. (2005). Rapid Prototyp. J..

[cit19] Halloran J. W., Tomeckova V., Gentry S., Das S., Cilino P., Yuan D., Guo R., Rudraraju A., Shao P., Wu T., Alabi T. R., Baker W., Legdzina D., Wolski D., Zimbeck W. R., Long D. (2011). J. Eur. Ceram. Soc..

[cit20] Tang H.-H., Chiu M.-L., Yen H.-C. (2011). J. Eur. Ceram. Soc..

[cit21] Salmoria G. V., Paggi R. A., Lago A., Beal V. E. (2011). Polym. Test..

[cit22] Slavko D. and Matic K., Annals of AAAM & Proceedings, 2010, p. 1527

[cit23] Wong K. V., Hernandez A. (2012). ISRN Mech. Eng..

[cit24] Murr L. E., Gaytan S. M., Ramirez D. A., Martinez E., Hernandez J., Amato K. N., Shindo P. W., Medina F. R., Wicker R. B. (2012). J. Mater. Sci. Technol..

[cit25] Tang H. P., Wang Q. B., Yang G. Y., Gu J., Liu N., Jia L., Qian M. (2016). JOM.

[cit26] Murr L. E., Martinez E., Amato K. N., Gaytan S. M., Hernandez J., Ramirez D. A., Shindo P. W., Medina F., Wicker R. B. (2012). J. Mater. Res. Technol..

[cit27] Murr L.E., Martinez E., Pan X. M., Gaytan S. M., Castro J. A., Terrazas C. A., Medina F., Wicker R. B., Abbott D. H. (2013). Acta Mater..

[cit28] Ramsperger M., Singer R. F., Körner C. (2016). Metall. Mater. Trans. A.

[cit29] Helmer H. E., Körner C., Singer R. F. (2014). J. Mater. Res..

[cit30] Monroy K., Delgado J., Ciurana J. (2013). Procedia Eng..

[cit31] Sachs E. M. , Haggerty J. S., Cima M. J. and Williams P. A., Three dimentional printing techniques, *US Pat.*, 5,340,656, 1994

[cit32] Lu K., Reynolds W. T. (2008). Powder Technol..

[cit33] Seitz H., Rieder W., Irsen S., Leukers B., Tille C. (2005). J. Biomed. Mater. Res., Part B.

[cit34] Godlinski D., Morvan S. (2005). Mater. Sci. Forum.

[cit35] Kim S. S., Utsunomiya H., Koski J. A., Wu B. M., Cima M. J., Sohn J., Mukai K., Griffith L. G., Vacanti J. P. (1998). Ann. Surg..

[cit36] Tay B. Y., Zhang S. X., Myint M. H., Ng F. L., Chandrasekaran M., Tan L. K. A. (2007). J. Mater. Process. Technol..

[cit37] Sirringhaus H., Shimoda T. (2011). MRS Bull..

[cit38] Ebert J., Özkol E., Zeichner A., Uibel K., Weiss Ö., Koops U., Telle R., Fischer H. (2009). J. Dent. Res..

[cit39] Meteyer S., Xu X., Perry N., Zhao Y. F. (2014). Procedia CIRP.

[cit40] Do T., Kwon P., Shin C. S. (2017). Int. J. Mach. Tools Manuf..

[cit41] Dilip J. J. S., Miyanaji H., Lassell A., Starr T. L., Stucker B. (2017). Def. Technol..

[cit42] Caputo M. P., Solomon C. V. (2017). Mater. Lett..

[cit43] Gaytan S. M., Cadena M. A., Karim H., Delfin D., Lin Y., Espalin D., MacDonald E., Wicker R. B. (2015). Ceram. Int..

[cit44] Hong D., Chou D.-T., Velikokhatnyi O. I., Roy A., Lee B., Swink I., Issaev I., Kuhn H. A., Kumta P. N. (2016). Acta Biomater..

[cit45] Gibson I. , Rosen D. and Stucker B., Additive Manufacturing Technologies: 3D Printing, Rapid Prototyping, and Direct Digital Manufacturing, Springer, New York, 2014

[cit46] Waller M. A., Fawcett S. E. (2014). J. Bus. Logist..

[cit47] Tofail S. A. M., Koumoulos E. P., Bandyopadhyay A., Bose S., O'Donoghue L., Charitidis C. (2018). Mater. Today.

[cit48] Sorrell S. (2010). Sustainability.

[cit49] Conti J. J. , Annual Energy Outlook 2012 with projections to 2035, U.S. Energy Information Administration (EIA), DOE/EIA-0383, June 2012

[cit50] Huang Y. and Leu M. C., Frontiers of Additive Manufacturing Research and Education, Research and Education Report of NSF Additive Manufacturing Workshop, 2014, pp. 1–35

[cit51] Karakoc T. H. , Ozerdem M. B., Sogut M. Z., Colpan C. O., Altuntas O. and Açıkkalp E., Sustainable Aviation: Energy and Environmental Issues, Springer, 2016

[cit52] Hasan S. and Rennie A. E. W., The application of Rapid Manufacturing technologies in the spare parts industry, in Nineteenth Annual International Solid Freeform Fabrication (SFF) Symposium, Austin, TX, USA, 4–8 Aug 2008

[cit53] Yang L. , Hsu K., Baughman B., Godfrey D., Medina F., Menon M. and Wiener S., in Additive Manufacturing of Metals: The Technology, Materials, Design and Production, Springer International Publishing, Cham, 2017, pp. 161–168, 10.1007/978-3-319-55128-9_6

[cit54] Liu R. , Wang Z., Sparks T., Liou F. and Newkirk J., in Laser Additive Manufacturing, Woodhead Publishing, 2017, pp. 351–371, 10.1016/B978-0-08-100433-3.00013-0

[cit55] Millsaps B. B. , Gulf of Mexico: Shell Uses 3D Printed Prototype for Very Complex Planning in Stones Deepwater Project, https://3dprint.com/130436/shell-3d-printed-prototyping, accessed 2017-09-22

[cit56] Boman K. , What Kind of Potential Does 3D Printing Hold for Oil, Gas?, http://www.rigzone.com/news/oil_gas/a/141296/What_Kind_of_Potential_Does_3D_Printing_Hold_for_Oil_Gas/?pgNum=0, accessed 2017-09-22

[cit57] Jan Holmström J. P., Tuomi J., Walter M. (2010). Journal of Manufacturing Technology Management.

[cit58] The opportunities for additive manufacturing in the energy and marine industries, Technology, Lloyd’s Register Technology, 2016

[cit59] Huang S. H., Liu P., Mokasdar A., Hou L. (2013). Int. J. Adv. Manuf. Technol..

[cit60] World’s first metal additively manufactured ship propeller nears completion, http://www.metal-am.com/worlds-first-metal-additively-manufactured-ship-propeller-nears-completion/, (accessed 2017-09-22)

[cit61] Maersk looks into on-ship 3D printing for tanker repair, (accessed 2017-09-22), https://youtu.be/vM6pzeDHnng

[cit62] Sharma H. , Assessing the Opportunity of Additive Manufacturing for the Oil and Gas Industry, Lux Research Inc, 2016

[cit63] H. Sharma, Lucrative Use For 3-D Printing In Oil And Gas Industry, https://www.epmag.com/lucrative-use-3-d-printing-oil-and-gas-industry-1483551#p=3, (accessed 2017-09-22)

[cit64] J. Snyder, The next revolution: how 3-D printing is transforming the energy sector, https://www.albertaoilmagazine.com/2014/02/3d-printing-energy-sector/)

[cit65] Gausemeier J. , Echterhoff N. and Wall M., Analysis of Promising Industries, Heinz Nixdorf Institute, University of Paderborn Product Engineering, Paderborn, 2011, p. 14

[cit66] Harris I. D. , Development and implementation of metals additive manufacturing, DOT International, New Orleans, LA, 2011

[cit67] Huang Y. , Liang X. and Azarmi F., in Pipelines 2014: From Underground to the Forefront of Innovation and Sustainability, 2014, pp. 1502–1511

[cit68] Monaghan T., Capel A. J., Christie S. D., Harris R. A., Friel R. J. (2015). Composites, Part A.

[cit69] Maier R. R. J., MacPherson W. N., Barton J. S., Carne M., Swan M., Sharma J. N., Futter S. K., Knox D. A., Jones B. J. S., McCulloch S. (2013). IEEE Sens. J..

[cit70] Gao W. , Zhang Y., Ramanujan D., Ramani K., Chen Y., Williams C. B., Wang C. C., Shin Y. C., Zhang S. and Zavattieri P. D., Computer-Aided Design, 2015, 69, pp. 65–89

[cit71] Cham J. G., Bailey S. A., Clark J. E., Full R. J., Cutkosky M. R. (2002). Int. J. Rob. Res..

[cit72] De Laurentis K. J. , Kong F. F. and Mavroidis C., 27th Biennial Mechanisms and Robotics Conference, 2002, 5, pp. 1239–1245

[cit73] Kataria A., Rosen D. W. (2001). Rapid Prototyp. J..

[cit74] Cham J. G. , Pruitt B. L., Cutkosky M. R., Binnard M., Weiss L. E. and Neplotnik G., Layered manufacturing with embedded components: process planning and issues, ASME proceedings, DETC 99, Las Vegas, Nevada, September 12–15, 1999

[cit75] Siggard E. J. , Madhusoodanan A. S., Stucker B. and Eames B., Structurally Embedded Electrical Systems Using Ultrasonic Consolidation (UC), 2006, pp. 70–83, https://public/SFF/SFF2006Proceedings/

[cit76] Zhao X., Pan Y., Zhou C., Chen Y., Wang C. C. L. (2013). J. Manuf. Process.

[cit77] Aguilera E. , Ramos J., Espalin D., Cedillos F., Muse D., Wicker R. and MacDonald E., in Proceedings of Solid Freeform Fabrication Symposium, 2013, pp. 950–961

[cit78] Meisel N. A., Elliott A. M., Williams C. B. (2015). J. Intell. Material Syst. Struct..

[cit79] Sbriglia L. R. , Baker A. M., Thompson J. M., Morgan R. V., Wachtor A. J. and Bernardin J. D., in Topics in Modal Analysis & Testing, Springer, 2016, vol. 10, pp. 205–214

[cit80] Tibbits S. (2014). Architectural Design.

[cit81] H. Bendemra, The future of 3D printing lies in space and with an extra dimension, https://theconversation.com/the-future-of-3d-printing-lies-in-space-and-with-an-extra-dimension-27267, (accessed 2017-09-22)

[cit82] Holweg M. , The limits of 3D printing, Harvard Business Review, Harvard Business Publishing, Boston, MA, 4th August 2015, https://hbr.org/2015/06/the-limits-of-3d-printing

[cit83] Piili H., Happonen A., Väistö T., Venkataramanan V., Partanen J., Salminen A. (2015). Phys. Procedia.

[cit84] Thomas D. S., Gilbert S. W. (2014). NIST Spec. Publ..

[cit85] Toshiba and Toshiba Machine Develop 3D Metal Printer, https://www.toshiba.co.jp/about/press/2015_11/pr2501.htm, 2017-09-22)

[cit86] First Additively Manufactured Part for Oil and Gas Certified by Lloyd’s Register, http://additivemanufacturing.com/2017/09/05/first-additively-manufactured-part-for-oil-and-gas-certified-by-lloyds-register/, (accessed 2017-09-22)

